# What Has Changed in the Behaviors of the Public After the COVID-19 Pandemic? A Cross-Sectional Study From the Saudi Community Perspective

**DOI:** 10.3389/fpubh.2022.723229

**Published:** 2022-03-21

**Authors:** Syed Wajid, Sana Samreen, Ibrahim Sales, Ghada Bawazeer, Mansour Adam Mahmoud, Majidah A. Aljohani

**Affiliations:** ^1^Department of Clinical Pharmacy, College of Pharmacy, King Saud University, Riyadh, Saudi Arabia; ^2^Aurobindo College of Pharmaceutical Science, Warangal, India; ^3^Department of Clinical and Hospital Pharmacy, College of Pharmacy, Taibah University, Medina, Saudi Arabia; ^4^Pharmaceutical Care Division, King Saud Medical City, Riyadh, Saudi Arabia

**Keywords:** COVID-19, social behavior, face mask, health practice, precautions

## Abstract

**Background:**

Coronavirus disease-2019 (COVID-19) is caused by severe acute respiratory syndrome-coronavirus (SARS-CoV) and represents a major public health threat that aggressively promotes increased morbidity and mortality. Changes in public behavior were more common during the pandemic to protect against the infection. Suboptimal behavioral practices for a specific disease would increase the susceptibility of the public to infection. This study aimed to determine changes in behaviors of the general public during the COVID-19 pandemic.

**Methods:**

A cross-sectional study was conducted using an online questionnaire survey to determine the extent of public behavioral changes in Saudi Arabia during the COVID-19 pandemic. Data were collected with a self-reported survey, and analysis was conducted using Statistical Package for the Social Sciences version 26 (SPSS). A chi-square test was performed to determine the association among variables. A *P* ≤ 0.05 was considered statistically significant.

**Results:**

Of 348 respondents, 244 (70.1%) were male, and 270 (77.6%) had a university degree. Approximately 48% used social media to seek COVID-19 information, and 36% (*n* = 124) avoided large gatherings. Approximately one-fourth of the respondents always avoided public transportation, while 65.8% reported avoiding traveling to infected areas. Of the participants, 33% always washed their hands, while 36% always used an alcohol-based sanitizer. There was a significant association between age group and employment status with respect to hand washing frequency (*p* < 0.05). There was also a significant association between age group (*p* < 0.0001) and employment status and wearing of face masks (*p* < 0.048).

**Conclusion:**

This study highlights changes in the public's behaviors in Saudi Arabia during the COVID-19 pandemic for protection against the infection and reports acceptable preventative practices against COVID-19 in the Saudi community. Furthermore, continuous awareness of recommended protective measures for COVID-19 is still warranted.

## Background

The recent viral pandemic known as coronavirus disease 2019 (COVID-19) caused by SARS-CoV, and the colossal impact on morbidity and mortality worldwide resulted in unprecedented changes both on the individual and global scales (1, 2). The virus, which was first identified in Wuhan, China and soon resulted in pandemic all over the world, manifests as severe acute respiratory syndrome symptoms that are not only extremely contagious but also life threatening (3). Previous studies have revealed that COVID-19 spreads through direct or indirect contact with an infected person or infected person's droplets from coughing, sneezing, or contaminated surfaces (4, 5). Depending on the type of surface, this virus has been reported to live for up to five consecutive days (6, 7). The World Health Organization (WHO) declared COVID-19 as a pandemic on March 11, 2020 (8). Until a few months ago, there was no available vaccine to protect the public, and preventative measures were mandated in most parts of the world to curb the spread of the virus (8). The WHO issued several guidelines about safety and preventive measures to contain the COVID-19 pandemic as preventative and treatment modalities were explored and investigated (8).

The WHO confirmed that 117,332,262 cases of COVID-19 were reported globally, and that ~2,605,356 deaths were reported globally on March 11, 2021 (8). Of the globally reported cases of COVID-19, ~24.5% were found in the United States, followed by India with 9.6%, Brazil with 9.41%, 3.7% in the Russian Federation, 0.17% in Kuwait, 0.14% in Qatar, and 0.32% in Saudi Arabia (9). The WHO and the Ministry of Health in Saudi Arabia established guidelines to control and prevent infection transmission including hand washing and/or the use of sanitizers, wearing face masks, limiting or controlling social activities, maintaining social distance, and avoiding contact with wild animals (10). More recently, the vaccine was introduced in the pharmaceutical market including in Saudi Arabia for the possible prevention of COVID-19 (11). However, healthcare bodies, such as the Centers for Disease Control and Prevention and the WHO, suggested to consider self-management and follow-up with infection prevention measures.

Preliminary literature reported that the gap or lack of knowledge of chronic diseases can increase morbidity and mortality (12). However, a number of studies evaluated the knowledge, attitudes, and practice on previous pandemics, such as malaria (13), intestinal parasitic infection (14), and influenza (15, 16). Until now, there are still limited studies that evaluated behavioral changes toward COVID-19 from a community perspective. Published studies reported that adequate behavioral practice and strict preventative measures would greatly influence morbidity and mortality rates, which in turn would help in controlling the spread of the disease (12, 17, 18). Implementing behaviors and taking precautionary measures during outbreaks and epidemics in a timely manner would help communities face upcoming epidemics in the future (19). Therefore, this study aimed to determine changes in behaviors of the general public in Saudi Arabia during the COVID-19 pandemic.

## Materials and Methods

An online internet-based non-interventional cross-sectional survey was conducted over a period of 2 months from October to December 2020 using Google Forms^®^ to collect data on the knowledge, protective measures, and sources of information about COVID-19 in Saudi Arabia. This study included adults aged ≥ 18 years who could read and understand the Arabic language.

### Ethical Approval

This investigation was carried out according to the guidelines of the Checklist for Reporting Results of Internet E-Surveys; also, the study followed the guidelines of the Declaration of Helsinki (20). Ethical approval was granted by the institutional review board of the College of Medicine, King Saud University, Riyadh, Saudi Arabia (E-20-5480). Prior to participating in the questionnaire, participants were provided an electronic consent form with a short introductory paragraph that stated the objective of the research and its importance. An additional statement indicating that participation in the research was voluntary and that participants had the full right to withdraw from the study at any point of time was also provided. There were no participant-specific identifiers collected and the participants were also assured that their data would only be used for research purposes, and that confidentiality of the data would be maintained. The participants were assured that there were no risks associated with their participation in this study. Moreover, the participants provided informed consent before answering the survey questions and were requested to provide authentic answers.

### Sample Size Calculation

This was a non-intervention-based research survey that measured changes in t behaviors of the general public after the COVID-19 pandemic, We estimated a total population size of 100,000. At 95% confidence interval with a 5% margin of error, the calculated sample size using Morgan's table was 384 (21).

### Development and Distribution of the Survey Instrument

The questionnaires used in this study were adopted from previous published studies (12, 18). The survey instrument was divided into four sections with a total of 20 items measuring changes in aspects of social behaviors and preventative measures of the general public after the COVID-19 pandemic. A pilot study revealed that surveys could be completed in <10 min. The first section of the study questionnaire obtained the respondents' demographic profile (age, sex, nationality, employment, education, and awareness about COVID-19. The second section contained six items about participant sources of COVID-19 information and used a four-point Likert scale (always/rarely). The third section of the study questionnaire consisted of three questions to rate practices of social behavior toward COVID-19 using a five-point Likert scale. The last part of the study instrument consisted of questions related to preventative measures and hygiene practices during the COVID-19 pandemic and used a five-point Likert scale (always/never). The survey questionnaires were translated into the Arabic language using the forward and backward translation procedure (22). Before using the questionnaires for data collection, the survey instrument was subjected to face and content validation by three selected senior faculty members from King Saud University College of Pharmacy to assess its readability and validity for clarity, significance, and acceptability. Modifications and refinements were made as per the received comments to better understand and organize the sequence of questions.

The final study tool used in data collection was using Google Forms^®^ through social media platforms, including WhatsApp^®^, Twitter^®^, and Facebook^®^. To achieve the required sample size, random and representative sample technique was used during the study period, and we followed several strategies. We first targeted our professional and personal networks. We expressed to them the importance of the study through personal messages and encouraged them to complete and forward the study questionnaires in their circles (personal and social). We also performed snowball sampling to achieve the required sample size (23). A total of 500 participants were targeted to achieve desired responses.

### Data Analysis

Data were initially collected in an Excel sheet and analyzed using SPSS version 24. Descriptive statistics, such as numbers and percentages, were used. A chi-square test was performed to investigate the level of association among study variables. A *p* < 0.05 was considered statistically significant.

## Results

A total of 348 respondents completed and returned the questionnaire by giving a response rate of (*n* = 500; 69%). More than two-thirds [244 (70.1%)] of the participants were male. Moreover, 134 (38.5%) were aged between 18 and 25 years, while 113 (32.5%) were aged between 31 and 40 years. Majority of the participants [230 (66.1%)] were Saudi nationals, while 270 (77.6%) had a university degree. More than half [203 (58.3%)] were employed. Most of the participants heard or had knowledge of COVID-19. Detailed responses of the participants are presented in Table 1. Slightly less than half of the participants [166 (47.7%)] claimed that they always use social media to obtain COVID-19 information, and 156 (44.8%) of the participants rarely used printed materials. Of the surveyed participants, approximately 21% (*n* = 72) used government websites, while 13.2% (*n* = 46) followed a family member's or a friend's advice. Details of the responses of resources used for COVID-19 are shown in Table 2.

**Table 1:** Participant (*n* = 348) demographics and professional characteristics.

**Parameters**	**Number (*n*)**	**Percentage (%)**
**Gender**		
Male Female	244 104	70.1 29.9
**Age (years)**		
18–25 26–30 31–40 > 40	134 33 113 68	38.5 9.5 32.5 19.5
**Nationality**		
Saudi Non-Saudi	230 118	66.1 33.9
**Educational level**		
Below university level University	78 270	22.4 77.6
**Employment status**		
Employed Unemployed	203 145	58.3 41.7
**Have you heard of COVID-19?**		
Yes No	344 4	98.9 1.1

**Table 2:** Sources of information on coronavirus disease-2019 (COVID-19) (*n* = 348).

**Parameters**	**Always**	**Often**	**Sometimes**	**Rarely**
Printed material	47 (13.5)	52 (14.9)	93 (26.7)	156 (44.8)
Social media (Facebook, Twitter, WhatsApp, YouTube, Instagram)	166 (47.7)	128 (36.8)	47 (13.5)	7 (2.0)
Official government websites	72 (20.7)	110 (31.6)	113 (32.5)	53 (15.2)
Radio	7 (2.0)	13 (3.7)	73 (21)	255 (73.3)
Television	27 (7.8)	33 (9.5)	137 (39.4)	151 (43.4)
Family member, colleague, or friend (4)	46 (13.2)	95 (27.3)	170 (48.9)	37 (10.6)

The current findings also revealed a strong relationship between age group and avoiding those who are sneezing or coughing (*p* = 0.0001), touching the face, mouth, eye, and nose (0.0001), and not spitting on the ground (*p* = 0.0001). Furthermore, there was a significant relationship between wearing gloves and age group (*p* = 0.0001), employment status (*p* = 0.048), and gender (*p* = 0.048). The gender and hygiene practice factors had no significant relationship (0.005) (Table 3).

**Table 3:** Participant responses toward (*n* = 348) preventative measures and hygiene practice during the COVID-19 pandemic.

**No**	**Parameters**	**Always**	**Frequently**	**Rarely**	**Occasionally**	**Never**
Q1	How often do you avoid people sneezing or coughing?	150 (43.1)	152 (43.7)	13 (3.7)	25 (7.2)	8 (2.3)
Q2	How often do you avoid touching the face, mouth, eye, and nose?	15 (4.3)	98 (28.2)	66 (19)	145 (41.7)	24 (6.9)
Q3	How often do you wash hands?	115 (33)	144 (41.4)	4 (1.1)	76 (21.8)	9 (2.6)
Q4	How often do you use alcohol-based disinfectant?	123 (35.3)	134 (38.5)	22 (6.3)	61 (17.5)	8 (2.3)
Q5	How often do you avoid spitting on the ground?	240 (69)	58 (16.7)	14 (4.0)	11 (3.2)	25 (7.2)
Q6	How often do you wear a mask?	250 (71.8)	71 (20.4)	5 (1.4)	15 (4.3)	7 (2.0)
Q7	How often do you wear gloves?	9 (2.6)	29 (8.3)	101 (29)	97 (27.9)	112 (32.3)

Regarding social behaviors of the participants, approximately 36% (*n* = 124) avoided large gatherings, while 15 (4.3%) never avoided large gatherings. Although 91 (26.1%) participants avoided public transportation, approximately 12% never did. Majority of the participants (*n* = 229; 65.8%) agreed that they always avoided traveling to infected areas. There was a significant association between age (*p* = 0.0001) and employment status with respect to large gatherings or public transportation (*p* = 0.0001) (Table 4). Moreover, there was a significant association between gender and age with respect to avoiding infected areas (*p* < 0.05) (Table 4).

**Table 4:** Association between demographics and social behaviors of the participants.

**No**	**Variables**	**Always**	**Frequently**	**Rarely**	**Occasionally**	**Never**	***P***
Avoid large gathering?	Sex						
	Male Female Age 18–25 26–30 31–40 <40 Employment Employed Unemployed	86(35.2) 38(36.5) 66(49.3) 13(39.4) 36(31.9) 9(13.2) 56(27.6) 68(46.9)	103(42.2) 43(41.3) 34(25.4) 9(27.3) 54(47.8) 49(72.1) 104(51.2) 42(29)	4(1.6) 3(2.9) 0(0) 3(9.1) 4(3.5) 0(0) 7(3.4) 0(0)	40(16.4) 16(15.4) 27(20.1) 0(0) 19(16.8) 10(14.7) 28(13.8) 28(19.3)	11(4.5) 4(3.8) 7(5.2) 8(24.2) 0(0) 0(0) 8(3.9) 7(4.8)	0.948 0.0001 0.0001
Public places/public transportation?	Sex						
	Male Female Age 18–25 26–30 31–40 <40 Employment Employed Unemployed	68(27.9) 23(22.1) 37(27.6) 9(27.3) 31(27.4) 14(20.6) 49(24.1) 42(29)	57(23.4) 20(19.2) 21(15.7) 6(18.2) 31(27.4) 19(27.9) 60(29.6) 17(11.7)	27(11.1) 23(22.1) 13(9.7) 13(39.4) 20(17.7) 4(5.9) 38(18.7) 12(8.3)	63(25.8) 26(25) 37(27.6) 3(9.1) 20(17.7) 29(42.6) 41(20.2) 48(33.1)	29(11.9) 12(11.5) 26(19.4) 2(6.1) 11(9.7) 2(2.9) 15(7.4) 26(17.9)	0.103 0.0001 0.0001
How often do you avoid travel to affected areas?	Sex						
	Male Female Age 18–25 26–30 31–40 <40 Employment Employed Unemployed	163(66.8) 66(63.5) 96(71.6) 18(54.5) 68(60.2) 47(69.1) 130(64) 99(68.3)	32(13.1) 5(4.8) 7(5.2) 4(12.1) 16(14.2) 10(14.7) 26(12.8) 11(7.6)	9(3.7) 7(6.7) 4(3.0) 5(15.2) 3(2.7) 4(5.9) 12(5.9) 4(2.8)	12(4.9) 8(7.7) 8(6) 3(9.1) 7(6.2) 2(2.9) 9(4.4) 11(7.6)	28(11.5) 18(17.3) 19(14.2) 3(9.1) 19(16.8) 5(7.4) 26(12.8) 20(13.8)	0.057 0.027 0.218

Regarding preventative measures, 150 (43.1%) of the participants avoided individuals who were sneezing or coughing, ~42% of the participants sometimes avoided touching the face, mouth, and nose, and only 4.3% always avoided it. One-third of the participants [115 (33%)] always washed their hands, while 144 (41.1%) frequently did. Approximately one-third of the participants (36%) always used alcohol-based sanitizers. With regard to wearing face masks, majority of the participants [250 (72%)] wore them always, while only 2% of the participants never wore face masks. There was a significant association between age group and employment status with respect to hand washing frequency (*p* < 0.05). Moreover, there was a significant association between age group and using alcohol-based sanitizers (*p* < 0.045). However, there was a significant association between age group (*p* < 0.0001) and employment status and wearing face masks (*p* < 0.048) (Tables 4, 5).

**Table 5:** Association between demographics and COVID-19 preventative measures.

**No**	**Parameters**	**Always**	**Frequently**	**Rarely**	**Occasionally**	**Never**	***p***
How often do you	Sex						
wash hands?	Male Female Age 18–25 26–30 31–40 <40 Employment Employed Unemployed	81(33.2) 34(32.7) 54(40.3) 4(12.1) 39(34.5) 18(26.5) 58(28.6) 57(39.3)	102(41.8) 42(40.4) 44(32.8) 20(60.6) 47(41.6) 33(48.5) 99(48.8) 45(31)	4(1.6) – 1(0.7) — 1(0.9) 2(2.9) 3(1.5) 1(0.7)	49(20.1) 27(26) 35(26.1) 4(12.1) 22(19.5) 15(22.1) 34(16.7) 42(29)	8(3.3) 1(1) — 5(15.2) 4(3.5) — 9(4.4) —	0.353 0.0001 0.0001
How often do you use disinfectant?	Sex						
	Male Female Age 18–25 26–30 31–40 <40 Employment Employed Unemployed	88(36.1) 35(33.7) 48(35.8) 10(30.3) 41(36.3) 24(35.3) 75(36.9) 48(33.1)	89(36.5) 45(43.3) 54(40.3) 13(39.4) 45(39.8) 22(32.4) 78(38.4) 56(38.6)	20(8.2) 2(1.9) 9(6.7) 6(18.2) 2(1.8) 5(7.4) 9(4.4) 13(19)	41(16.8) 20(19.2) 20(14.9) 2(6.1) 22(19.5) 17(25) 36(17.7) 25(17.2)	6(2.5) 2(1.9) 3(2.2) 2(6.1) 3(2.7) — 5(2.5) 3(2.1)	0.209 0.045 0.530
Wearing mask?	Sex						
	Male Female Age 18–25 26–30 31–40 <40 Employment Employed Unemployed	174(71.3) 76(73.1) 99(73.9) 24(72.7) 83(73.5) 44(64.7) 140(69) 110(75.9)	54(22.1) 17(16.3) 30(22.4) — 17(15) 24(35.3) 41(20.2) 30(20.7)	4(1.6) 1(1) — 2(6.1) 3(2.7) – 5(2.5) —	6(2.5) 9(8.7) 5(3.7) – 10(8.8) – 10(4.9) 5(3.4)	6(2.5) 1(1) — 7(21.2) — – 7(3.4) —	0.067 0.0001 0.048

## Discussion

In Saudi Arabia, the first COVID-19 case was reported on March 2, 2020. Shortly after the confirmation of the first COVID-19 case, the Saudi government began an official lockdown on March 9, 2020 (26). Furthermore, to prevent rapid transmission of the virus, similar to other countries, the Saudi government implemented regulations and measures including public and private travel restrictions locally and internationally, lockdowns and curfews, and household quarantines as means to control the epidemic of COVID-19 in the Kingdom of Saudi Arabia (25, 27, 28). Additionally, the Ministry of Health in Saudi Arabia imposed several infectious control measures similar to other international countries that included wearing masks, hand hygiene, and social distancing (29). Despite all of these efforts by healthcare authorities for the prevention or control of the COVID-19 pandemic, COVID-19 cases continue to rise worldwide, and reduction of virus transmission varies based on individual behaviors and knowledge of preventative measures.

Maintaining a healthy environment is possible only when all communities at both the national and international levels strictly adhere to guidelines prescribed by government authorities to combat the spread of the virus. In all of these guidelines, emphasis has been made on hand washing, avoiding crowded places, and wearing face masks (29). In this study, majority of the participants washed their hands using alcohol-based sanitizers. Our results were similar to those of a previous study during early stages of the pandemic by Almalki, who reported that 96.7% of the participants washed their hands as recommended by the Ministry of Health (30). However, our results were slightly better than those of a more recent study by Battineni et al., who reported that ~94% of seafarers washed their hands by rubbing at least 20 s with the use of sanitizers (31).

At the beginning of the COVID-19 pandemic, there was a significant concern about the virus' longevity on surfaces. Most guidelines emphasized avoiding touching surfaces, hand washing, using hand sanitizers, and social distancing (1–2 m) to prevent transmission (32). In this study, more than one-third (36%) of the participants always avoided large gatherings, and ~ 66% avoided traveling to infected areas. These results were inconsistent with the findings of a similar study on seafarers published by Battineni et al., who reported that 68% traveled to infected areas. Moreover, previous studies have suggested that traveling is more common, with either presence or absence of infection, particularly in Western countries (32).

Behaviors of an individual depend on their knowledge about the disease. This study showed that approximately 87% of the Saudi population always avoided individuals who are sneezing or coughing, and that 43% avoided touching their mouth, nose, and eyes. These findings suggest that hand washing behaviors were acceptable, with 75% of the respondents adhering to recommended hand washing recommendations. These results still showed lower rates than those in a previous study by Battineni et al., who reported that between 97 and 93% of respondents avoided touching their faces and being in close contact with individuals with flu-like symptoms, and that most of the respondents (94%) washed their hands (31). The difference in behaviors of this current study population might be due to the time of the questionnaire distribution (i.e., during later phases of the outbreak), extremely smaller number of cases reported by the Ministry of Health compared to those of other countries, and optimism that COVID-19 would be eradicated as the government launched the vaccine (24, 27, 33).

Regarding Saudi public behaviors toward wearing face masks and hand gloves, approximately 93% of the participants always wore a face mask. In contrast, the rate of wearing gloves was extremely low (10.9%). There is evidence that wearing masks helps in protecting the transmission of COVID-19 infection, including other droplets; however, another recent study by Yasser and Sultan reported that approximately 50% of participants wore a protective face mask when leaving home (23, 27). Moreover, this study found a significant association between wearing a face mask and washing hands with respect to age and employment status of the participants (*p* < 0.05). These results were similar to those of previous studies on an Iranian population and Dutch cyclists (24, 34). One study reported that male sex and adolescence were factors associated with unsafe behaviors during the pandemic period (35). However, in this study, the association between wearing masks and sex may be related to Islamic culture, where many women wore face veils when they are in public as a protective mask, which might be a significant factor for the association of wearing a mask and gender. Moreover, to the best of our knowledge, during this pandemic, due to high alertness and awareness by the government and fear of infection, most employed individuals who left their homes were observed to apply more hygienic practices, such washing hands, compared to unemployed individuals (31). However, these results show similar practices among employed and unemployed individuals with respect to hand washing frequency (*p* < 0.05) and use of face masks (*p* < 0.05). The results also revealed that there was a significant association between gender and age of the respondents and avoidance of large gatherings, traveling to public places/public transportation, and traveling to infected areas (*p* < 0.05). There are some limitations that should be mentioned regarding this study. The number of participants who completed this survey was less than the calculated sample size, and no information was collected from the respondents regarding their geographic locations in Saudi Arabia. These factors may limit the generalizability of the results.

## Conclusions

The results of this study concluded changes in public attitudes and behaviors with respect to wearing masks and hand washing practices. In addition, behaviors toward traveling also changed with respect to COVID-19 precautions for protection against the infection. Moreover, the results revealed acceptable preventative practices against the spread of COVID-19 in the Saudi community. Furthermore, awareness of appropriate behavioral practices about protective measures for COVID-19 is warranted.

## Data Availability Statement

The original contributions presented in the study are included in the article/supplementary material, further inquiries can be directed to the corresponding author.

## Ethics Statement

Ethical approval was granted by the Institutional Review Board of the College of Medicine, King Saud University, Riyadh, Saudi Arabia (E-20-5480). The patients/participants provided their written informed consent to participate in this study.

## Author Contributions

SW: conceptualization, data curation, formal analysis, and visualization. SS: writing (original draft preparation, review, and editing). IS: funding acquisition and manuscript review and editing. MM: formal analysis and review and editing. GB and MA: writing (review and editing). All authors have read and agreed to the published version of the manuscript.

## Conflict of Interest

The authors declare that the research was conducted in the absence of any commercial or financial relationships that could be construed as a potential conflict of interest.

## Publisher's Note

All claims expressed in this article are solely those of the authors and do not necessarily represent those of their affiliated organizations, or those of the publisher, the editors and the reviewers. Any product that may be evaluated in this article, or claim that may be made by its manufacturer, is not guaranteed or endorsed by the publisher.

## Abbreviations

COVID-19: coronavirus disease-2019
CHERRIES: Checklist for Reporting Results of Internet E-Surveys
WHO: World Health Organization
USA: United States of America
MERS-CoV: Middle East respiratory syndrome coronavirus
KSA: Kingdom of Saudi Arabia.
